# ∆Np63α inhibits Rac1 activation and cancer cell invasion through suppression of *PREX1*

**DOI:** 10.1038/s41420-023-01789-0

**Published:** 2024-01-08

**Authors:** Amjad A. Aljagthmi, Akshay Hira, Jin Zhang, Mariana Cooke, Marcelo G. Kazanietz, Madhavi P. Kadakia

**Affiliations:** 1 Department of Biochemistry and Molecular Biology, Boonshoft School of Medicine, Wright State University, 3640 Colonel Glenn Highway, Dayton, OH 45435 USA; 2 Department of Systems Pharmacology and Translational Therapeutics, Perelman School of Medicine, University of Pennsylvania, Philadelphia, PA 19104 USA

**Keywords:** Head and neck cancer, Oncogenes

## Abstract

ΔNp63α, a member of the p53 family of transcription factors, plays a critical role in maintaining the proliferative potential of stem cells in the stratified epithelium. Although ΔNp63α is considered an oncogene and is frequently overexpressed in squamous cell carcinoma, loss of ΔNp63α expression is associated with increased tumor cell invasion and metastasis. We recently identified a ΔNp63α/miR-320a/PKCγ signaling axis that regulates cancer cell invasion by inhibiting phosphorylation of the small GTPase Rac1, a master switch of cell motility that positively regulates cell invasion in multiple human cancers. In this study, we identified a novel mechanism by which ΔNp63α negatively regulates Rac1 activity, by inhibiting the expression of the Rac-specific Guanine Exchange Factor PREX1. ΔNp63α knockdown in multiple squamous cell carcinoma cell lines leads to increased Rac1 activation, which is abrogated by treatment with the Rac1 inhibitor NSC23766. Furthermore, ΔNp63α negatively regulates *PREX1* transcript and protein levels. Using a Rac-GEF activation assay, we also showed that ΔNp63α reduces the levels of active PREX1. The inhibition of the PREX1-Rac1 signaling axis by ΔNp63α leads to impaired cell invasion, thus establishing the functional relevance of this link. Our results elucidated a novel molecular mechanism by which ΔNp63α negatively affects cancer cell invasion and identifies the ΔNp63α/Rac1 axis as a potential target for metastasis.

## Introduction

ΔNp63α, the major isoform of p63 expressed in epithelial tissues, plays a dual role in cancer, both as an oncogene and as a suppressor of tumor metastasis [1–3]. While ΔNp63α is frequently overexpressed and promotes cell proliferation during the early stages of squamous cell carcinoma (SCC) [1, 4], loss of ΔNp63α promotes cancer metastasis and is associated with poor prognosis [5–8]. It is well-established that ΔNp63α reduces cancer cell invasiveness and prevents metastatic dissemination of different cancer cells [5, 6, 8, 9], however the precise mechanisms underlying these effects are poorly characterized.

The small GTPase Rac1 regulates multiple signaling pathways that control cytoskeleton organization, transcription, and cell proliferation. Deregulated Rac1 expression and/or activity is a common event in cancer, and has been associated with anchorage-independent growth, transformation, migration, and invasion [10, 11]. Rac1 is activated by guanine exchange factors (GEFs) that promote GDP/GTP exchange, and inactivated by GTPase activating proteins (GAPs). Rac1 hyperactivation in cancer cells is frequently caused by the oncogenic activation of upstream GEFs, or by the dysregulation of GEF expression or activity [12]. The Rac-GEF family comprises more than 40 members, with most of them (>30) belonging to the Dbl-like class, and a smaller subset corresponding to the DOCK family [13]. As a large family of multidomain proteins, Rac-GEFs have distinctive regulatory modes and display cell-type specific differences in expression. Rac-GEFs have been shown to regulate cell invasiveness and metastatic dissemination of cancer cells [13, 14]. Moreover, elevated expression of discrete Rac-GEFs has been associated with poor clinical outcome in several cancer types [13, 15, 16]. Among the pro-metastatic Rac-GEFs, Phosphatidylinositol-3,4,5-Trisphosphate Dependent Rac Exchange Factor 1 (PREX1) has been found to be highly expressed in many types of tumors, including melanoma, breast, prostate cancer and others [17]. Elevated PREX1 expression has been linked to migratory and invasive phenotypes of cancer cells, and conversely, PREX1 knockdown suppresses cell migration and invasion [18–22]. PREX1 is synergistically activated by PIP3 (a lipid product of PI3K) and Gβγ subunits of heterotrimeric G proteins [17, 23]. The pathways that regulate PREX1 expression and function are poorly understood, and its role in SCC has not been yet identified.

Here, we demonstrate that ΔNp63α acts as a negative regulator of Rac1 activation by suppressing the expression of PREX1. Our data provide critical insight into the nature of ΔNp63α effectors and underscore a novel regulatory signaling pathway for Rac1-mediated cancer cell invasion.

## Results

### ΔNp63α negatively regulates Rac1-GTP

**Fig. 1:**
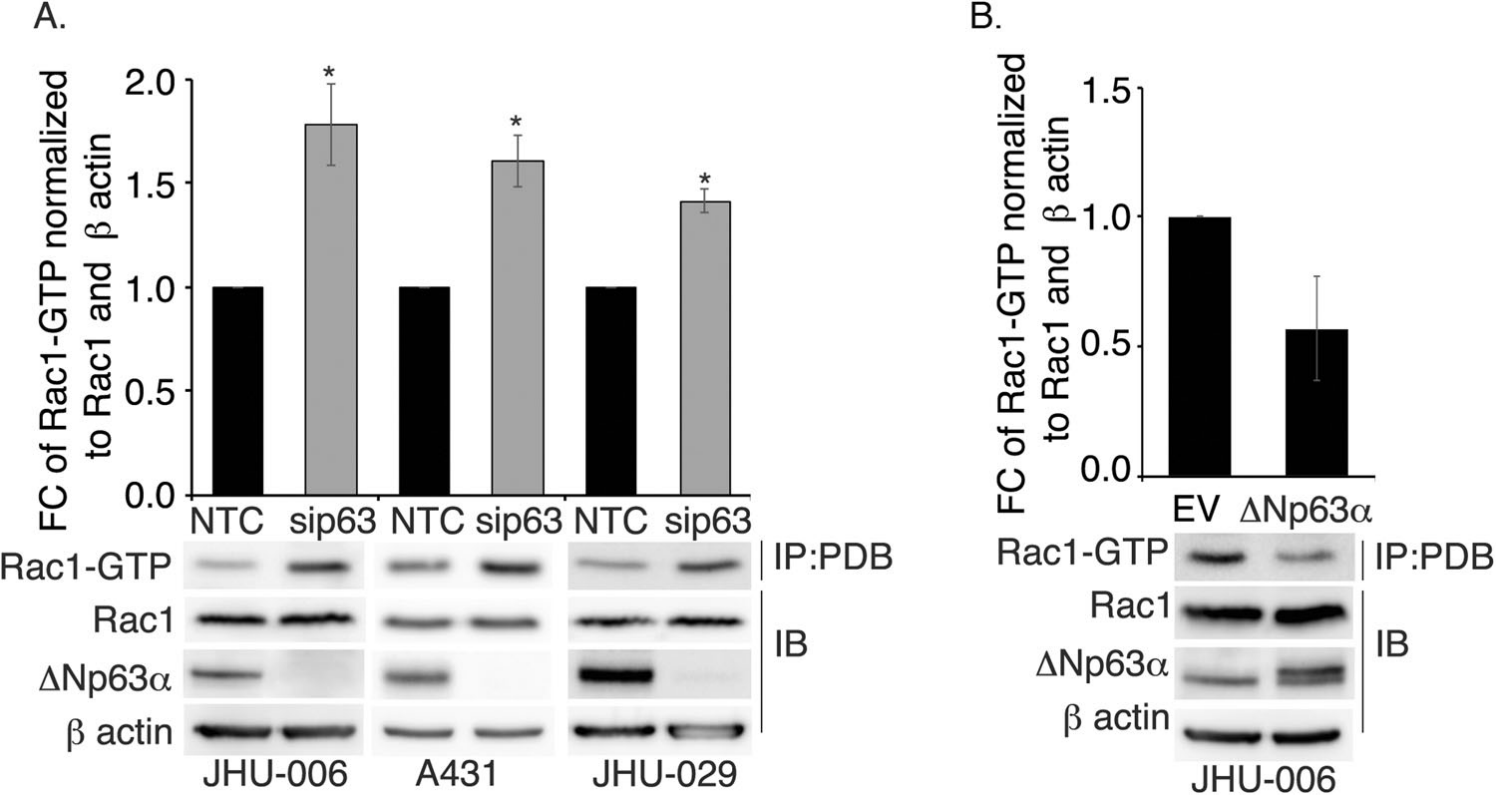
ΔNp63α negatively regulates Rac1-GTP in SCC cells. **A** JHU-006, A431, and JHU-029 cells were transfected with non-targeting control (NTC) siRNA or p63-targeted siRNA (sip63). **B** JHU-006 cells were transfected with empty vector (EV) or ΔNp63α-expression plasmid DNA. Whole-cell lysates of transfected cells were immunoprecipitated with p21-activated kinase (PAK) protein binding domain (PBD) followed by immunoblot with Rac1 antibody to detect Rac1-GTP, as described in Materials and Methods. Immunoblot analysis with the indicated antibodies is shown in the bottom panels. β-actin was used as a control to normalize for differences in total protein per lane. The relative abundance of Rac1-GTP was calculated by normalizing the total Rac1 and β-actin signal in the corresponding NTC (**A**) or EV control (**B**), and the resulting fold-changes are shown in the bar plots (top). Data are presented as mean ±1 S.E.M. Asterisks indicate *P* ≤ 0.05 relative to corresponding NTC.

To determine whether ΔNp63α inhibits the activation of Rac1, active Rac1 (Rac1-GTP) levels were measured using Rac1-GTP pull-down assay [24] in different SCC cell lines subjected to ΔNp63α knockdown (Fig. 1A). ΔNp63α knockdown caused a significant elevation in Rac1-GTP levels in A431 epidermal SCC cells as well as in JHU-006 and JHU-009 head and neck squamous cell carcinoma (HNSCC) cells. Consistent with our prior findings [25], total Rac1 protein was unaffected by ΔNp63α knockdown (Fig. 1A). Conversely, when ΔNp63α was overexpressed in JHU-006 cells, Rac1-GTP levels were lower than in control cells (EV, empty vector) (Fig. 1B). ∆Np63α protein knockdown and overexpression were confirmed by Western blot on total cell lysates (Fig. 1A, B, bottom panels). These data suggest that ΔNp63α negatively regulates the activation status of Rac1.

### ΔNp63α inhibits cancer cell invasion by impairing Rac1 activation

**Fig. 2:**
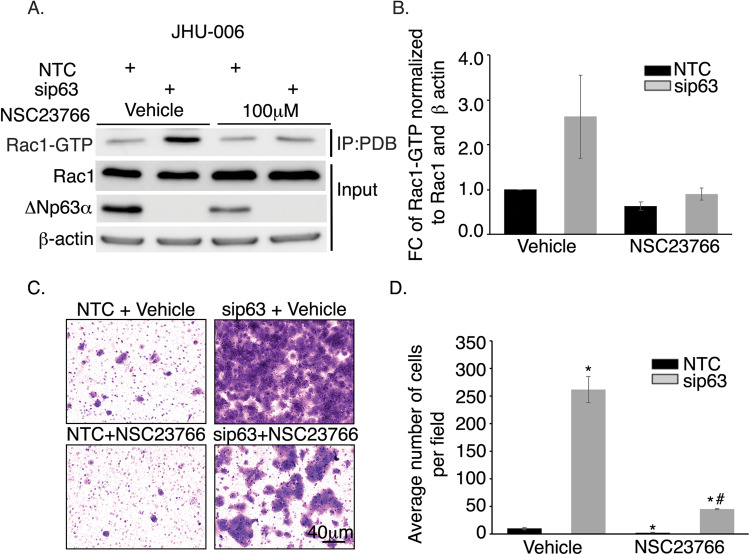
Rac1 inhibitor NSC23766 abrogates the effect of ∆Np63α knockdown on activation of Rac1 and cell invasion. **A** JHU-006 cells were transfected with non-targeting control (NTC) siRNA or p63-targeted siRNA (sip63), and transfected cells were treated with vehicle (water) or 100 μM NSC23766 for 24 h. Whole-cell lysates were immunoprecipitated with p21-activated kinase (PAK) protein binding domain (PBD) followed by immunoblot with Rac1 antibody to detect Rac1-GTP. Immunoblot analysis was performed with the indicated antibodies and β-actin was used as a loading control. **B** The fold-change (FC) in Rac1-GTP levels normalized to total Rac1 and β actin from n = 3 independent runs. **C** JHU-006 cells were transfected with NTC or sip63, and transfected cells were treated with vehicle or 100 µM NSC23766. After 24 h, an aliquot of 5.0 × 10^4^ cells was analyzed using the Matrigel cell invasion assay as shown in the representative experiment. **D** Invading cells were counted after 21 h. Error bars indicate mean ± SEM. Statistically significant values (*P* ≤ 0.05) relative to vehicle/NTC (*) or vehicle/sip63 (#) determined by mixed effects ANOVA are indicated.

Next, we examined the effect of NSC23766, a small molecule inhibitor that inhibits the interaction between the Rac1 Switch II domain and Rac-specific GEFs [26, 27], in JHU-006 cells on Rac1 activation caused by ∆Np63α knockdown. Notably, NSC23766 abrogated the elevation in Rac1-GTP levels resulting from ΔNp63α knockdown (Fig. 2A, B). ∆Np63α protein knockdown and the lack of an effect on total Rac1 levels was confirmed by Western blot as in Fig. 1 (Fig. 2A).

In order to determine the functional consequences of ∆Np63α on Rac1 activity, we assessed cell invasion by Matrigel transwell assay in JHU-006 cells. ΔNp63α knockdown significantly increased cell invasion relative to non-targeting control (NTC) cells (Fig. 2C, D). Conversely, NSC23766 treatment reduced cell invasion in both NTC and sip63 cells relative to corresponding vehicle-treated controls (Fig. 2C, D), suggesting ΔNp63α-dependent inhibition of Rac1-GTP levels leads to decreased cell invasiveness.

### ΔNp63α knockdown negatively regulates *PREX1* expression

**Fig. 3:**
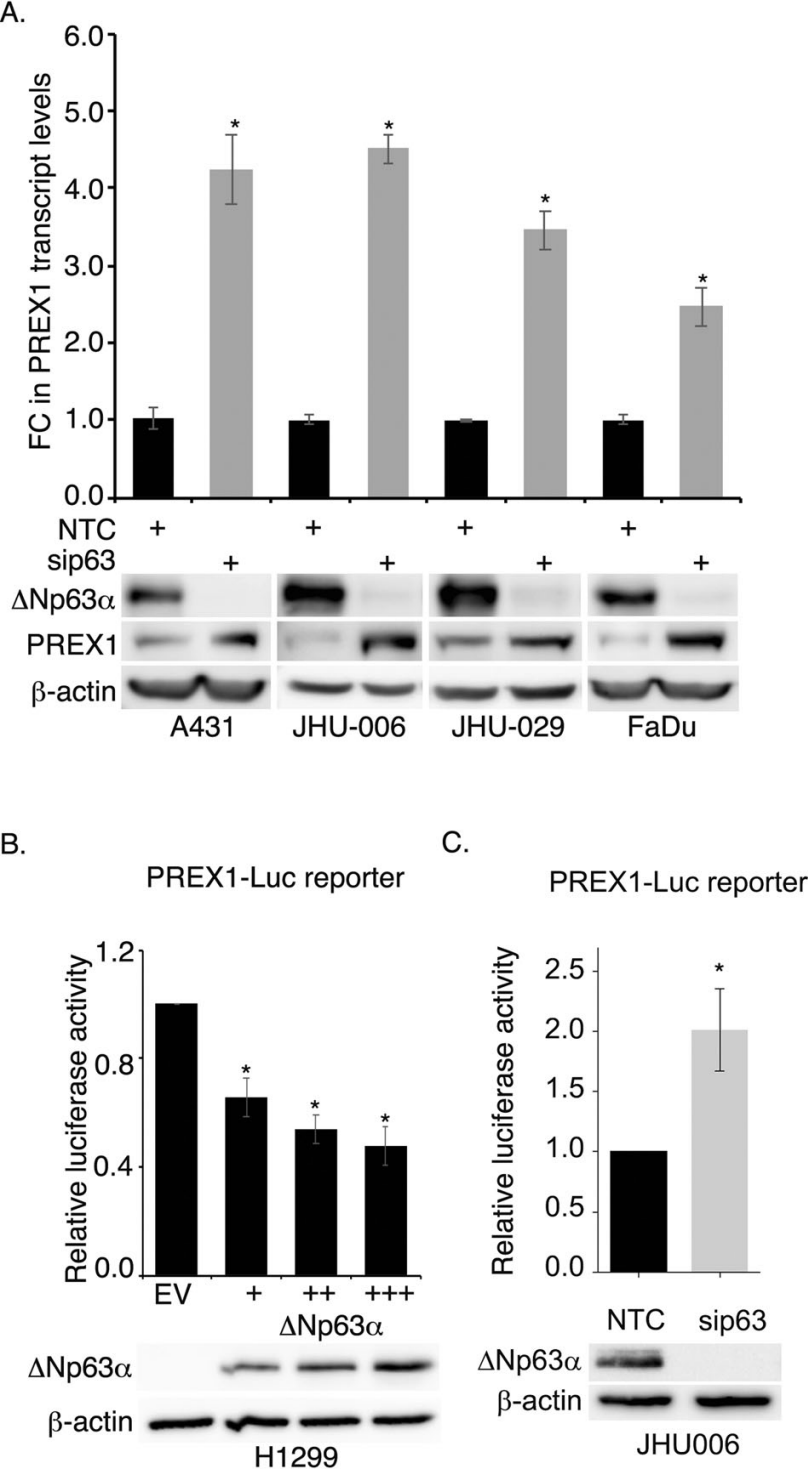
ΔNp63α negatively regulates *PREX1*. **A** A431, JHU-006, JHU-029, and FaDu cells were transfected with non-targeting control (NTC) siRNA or p63-targeted siRNA (sip63). 48 h after transfection, *PREX1* mRNA was quantified using TaqMan qRT-PCR (top panel). Values were normalized to the NTC control (black bar) and the fold-change (FC) in *PREX1* mRNA (gray bar) relative to NTC is shown in the top of panel A. Error bars represent ±1 SD. Statistically significant values (*P* ≤ 0.05) relative to corresponding NTC are indicated with an asterisk. Immunoblot of ∆Np63α and PREX1 protein are shown in the bottom panel. β-actin was used as a loading control. **B** H1299 cells were co-transfected with *PREX1*-Luc reporter plasmid DNA and either empty vector (EV) control or increasing concentrations of ΔNp63α expression plasmid. At 24 h after transfection, a dual luciferase assay was performed in triplicate. Relative luciferase units (RLU) were calculated from the ratio of Firefly luciferase activity to *Renilla* luciferase activity normalized to EV control. Values are shown as mean ±1 S.E.M. Statistically significant values (*P* ≤ 0.05) relative to EV controls are indicated with an asterisk. **C** JHU-006 cells were co-transfected with non-targeting control (NTC) siRNA or p63-targeted siRNA (sip63). After 24 h, cells were transfected with *PREX1*-Luc reporter plasmid. Following another 24 h, a dual luciferase assay was performed. Relative luciferase units (RLU) were calculated as the ratio of Firefly luciferase activity to *Renilla* luciferase activity and normalized to NTC control. Values are shown as mean ±S.E.M. Statistically significant values (*P* ≤ 0.05) relative to NTC controls are indicated with an asterisk. ∆Np63α protein and the β-actin loading control were analyzed by immunoblot (bottom).

Since Rac1 hyperactivation in cancer cells frequently correlates with elevated expression and/or activation of Rac1-GEFs [13], we speculated that the ∆Np63α-mediated reduction of Rac1 activity could be associated with a reduction in the levels of upstream Rac-GEFs. Since the Rac-GEF family comprises >40 members, we took advantage of a pre-designed Q-PCR array [28] to assess the effect of ΔNp63α knockdown on Rac-GEF expression in A431 cells. Two different ΔNp63α siRNA duplexes were used to minimize misinterpretation of results due to non-specific effects of RNAi. Our analysis revealed that several DOCK Rac-GEFs and Dbl-like Rac-GEFs were upregulated by ΔNp63α knockdown with both siRNA duplexes (Supplemental Fig. 1). Among the upregulated GEFs, PREX1 was chosen for subsequent analysis due to of its known pro-invasive role in cancer [20, 21]. The effect of ΔNp63α knockdown on PREX1 expression was examined in A431, JHU-006, JHU-029 and FaDu SCC cell lines. ΔNp63α knockdown led to a significant upregulation of *PREX1* mRNA (Fig. 3A, upper panel) and increased protein levels (Fig. 3A, *lower panel*), as determined by qRT-PCR and Western blot analysis, respectively. These results were also recapitulated in HaCaT cells [29], a non-tumorigenic human keratinocyte cell line, which showed a reduction in PREX1 mRNA with ΔNp63α knockdown (Supplemental Fig. 2). Taken together, these results indicate that ΔNp63α negatively regulates PREX1 transcript and protein levels in SCC.

Next, we wanted to determine the effect of ΔNp63α on the promoter activity of PREX1. A 2 kB fragment of the *PREX1* promoter (−2047 to −23 from the transcriptional start site) was cloned upstream of a luciferase reporter gene in the pGL3-Basic vector. The resulting pGL3-PREX1-Luc reporter plasmid [22] (*PREX1*-Luc) was co-transfected into p63 null H1299 cells with a *Renilla* luciferase plasmid (for normalization), along with either empty vector (control) or increasing concentrations of a ΔNp63α expression plasmid. Increasing concentrations of ΔNp63α, as confirmed by immunoblot, led to a dose-dependent reduction in luciferase reporter activity (Fig. 3B). Conversely, an increased luciferase reporter activity was observed upon knockdown of ΔNp63α in JHU-006 cells (Fig. 3C). ΔNp63α knockdown was confirmed by Western blotting. These results strongly support the findings that ΔNp63α negatively regulates PREX1 by inhibiting its transcription.

To investigate the mechanism by which ΔNp63α suppresses PREX1 transcription, we examined whether PREX1 is a direct transcriptional target of ΔNp63α. Therefore, the p63 ChIP-seq database GSE59827 [30, 31] was used to identify putative p63 binding sites in the *PREX1* promoter. This analysis predicted a p63 binding site located at chr20:48828385-48828404 with the sequence 5’- GCGCAGGCTCCTGCTTGCAG-3’. Next, a 229 bp fragment of the *PREX1* promoter containing the putative p63 binding site was cloned upstream of the luciferase reporter gene in pGL3 to generate the Δ*PREX1*-Luc reporter plasmid. Δ*PREX1*-Luc was co-transfected into p63 null H1299 cells together with either empty vector (control) or increasing concentrations of the expression plasmid encoding ΔNp63α. Dose-dependent expression of ΔNp63α caused a significant decrease in luciferase reporter activity (Supplemental Fig. 3A). As observed with the full-length PREX1 promoter (Fig. 3C), ΔNp63α knockdown led to an increase in the ΔPREX1 promoter activity, which was not affected when this binding site was mutated (Supplemental Fig. 3B). Moreover, ChIP analysis using p63 specific antibodies did not show binding of p63 to this binding site (data not shown). Taken together, these results suggest that the *PREX1* is not a direct transcriptional target of ΔNp63α.

### ΔNp63α knockdown increases Rac1 binding to activated PREX1

**Fig. 4:**
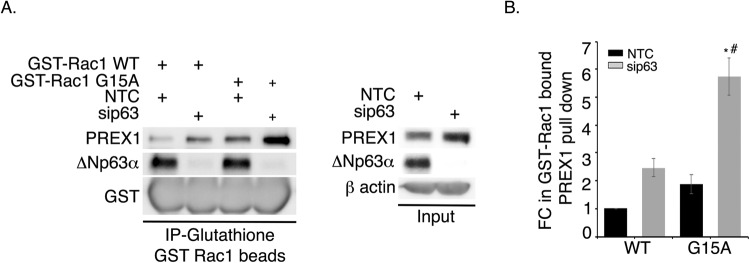
ΔNp63α knockdown increases active PREX1 binding to Rac1. **A** JHU-006 cells were transfected with non-targeting control siRNA (NTC) or p63-targeted siRNA (sip63). At 48 h after transfection, cells were harvested and lysates prepared for immunoprecipitation (IP) using wild-type (WT) or G15A GST-Rac1 beads. Immunoblot analysis of PREX1, p63, and GST in RAC1-GST IP samples (left), and whole-cell lysates (right) was performed. β-actin was used as a loading control (right). **B** The fold-change in PREX1-bound GST-Rac1 (WT or G15A) relative to WT/NTC or G15A/NTC, respectively, was calculated for cells transfected with NTC (black bars) or sip63 (gray bars). Error bars indicate mean ± SEM. Statistically significant values (*P* ≤ 0.05) relative to WT/NTC (*) or G15A/NTC (#) are indicated.

To determine whether modulation of ΔNp63α expression leads to changes in PREX1 activity, we used a pull-down assay for activated PREX1. We took advantage of the G15A-Rac1 “nucleotide-free” mutant, which binds poorly to GDP and GTP, and thus mimics the intermediate state that binds to active Rac-GEFs with high affinity [32]. PREX1 was immunoprecipitated from whole lysates of JHU-006 cells transiently transfected with either non-targeting control (NTC) or ΔNp63α siRNA, followed by incubation with either GST-WT-Rac1 or GST-G15A-Rac1 fusion proteins. This assay revealed that ∆Np63α knockdown increased binding of PREX1 to WT Rac1 relative to control cells (Fig. 4A, lane 2 vs. lane 1, Fig. 4B). A stronger effect was observed with the G15A-Rac1 mutant (Fig. 4A, lane 4 vs. lane 3, and Fig. 4B). Altogether, these results indicate that ΔNp63α knockdown not only increases the abundance of PREX1 transcript and protein levels, but also results in elevated PREX1 activity.

### Increased Rac1-GTP in ΔNp63α knockdown cells is dependent on PREX1

**Fig. 5:**
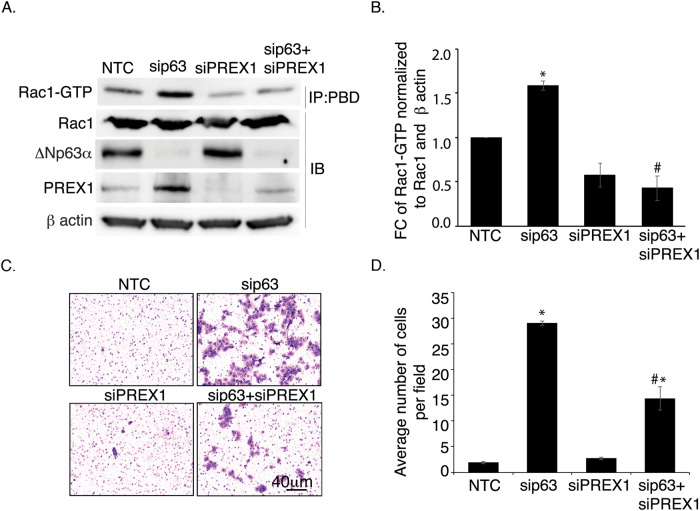
Knockdown of *PREX1* abrogates the effect of ΔNp63α knockdown on the activation of Rac1 and cell invasion. **A** JHU-006 cells were transfected with non-targeting control siRNA (NTC), p63-targeted siRNA (sip63), and/or *PREX1*-targeted siRNA (si*PREX1*) as indicated. At 48 h after transfection, cells were harvested and Rac1-GTP was quantified using Rac1 pull-down assay, followed by immunoblot with antibodies to p63 and Rac1, as indicated. β-actin was used as a loading control. **B** The fold-change (FC) in Rac1-GTP levels normalized to total Rac1 and the β-actin loading control. **C** JHU-006 cells were transfected with NTC, sip63, si*PREX1*, or sip63 and si*PREX1*. At 48 h after transfection, an aliquot containing 5.0 × 10^4^ cells was analyzed using the Matrigel cell invasion assay as shown in the representative experiment. **D** Invading cells per field were counted after 21 h. Statistically significant values (*P* ≤ 0.05) relative to NTC or sip63 as determined by mixed effects ANOVA are indicated with an * or a #, respectively.

Based on the above results, we speculated that silencing PREX1 in ΔNp63α knockdown cells should reduce Rac1 activation. Therefore, JHU-006 cells were transiently transfected with non-targeting control (NTC) or siRNA to PREX1 (siPREX1) and/or ΔNp63α (sip63). Knockdown of PREX1 and/or p63 were confirmed by immunoblot. Consistent with Figs. 1A and 3A, ΔNp63α knockdown increased PREX1 protein and Rac1-GTP levels (Fig. 5A, lane 2 vs. lane 1, and Fig. 5B), whereas notably, PREX1 knockdown reduced PREX1 and Rac1-GTP levels relative to NTC control cells (Fig. 5A, lane 3 vs. lane 1, and Fig. 5B). Importantly, in ΔNp63α and PREX1 double knockdown cells, Rac1-GTP levels were significantly lower than in cells with knockdown of ΔNp63α alone (Fig. 5A, lane 4 vs. lane 2, and Fig. 5B). These results indicate that ΔNp63α reduces Rac1-GTP levels by negatively regulating the expression of PREX1.

### *PREX1 silencing* reduces invasion in ΔNp63α knockdown cells

Since *PREX1* levels positively correlate with cell migration and invasion [17–21, 33], we next determined whether ΔNp63α-dependent repression of *PREX1* inhibits cell invasion. A Matrigel-based invasion assay was performed using JHU-006 cells transiently transfected with ΔNp63α and/or *PREX1* siRNA. As expected, ΔNp63α knockdown increased cell invasion relative to NTC. Interestingly, while *PREX1* RNAi had no significant effect on cell invasiveness (Fig. 5C and D), ΔNp63α and *PREX1* double knockdown cells were less invasive than ΔNp63α knockdown cells, indicating that knockdown of *PREX1* reversed the effect of ΔNp63α knockdown on cell invasion. Altogether, these data demonstrate that ΔNp63α inhibits cell invasion by negatively regulating expression of *PREX1*.

## Discussion

ΔNp63α plays a dual role in cancer. As a strong oncogenic protein in SCC, ΔNp63α promotes cell survival and angiogenesis, suppresses apoptosis, and is associated with poor prognosis [34–37]. Conversely, loss of ΔNp63α correlates with increased invasiveness and metastatic capacity of SCC cells [5, 6, 8]. Moreover, decreased ΔNp63α expression upregulates epithelial-to-mesenchymal transition (EMT) genes in cell culture and promotes metastatic spread in mice [6, 9, 38]. The precise mechanisms by which ΔNp63α inhibits cancer cell invasion have been poorly characterized. We recently showed that ΔNp63α reduces Rac1 signaling by inhibiting protein kinase C γ (PKCγ), which in turn inhibits cancer cell invasion [25]. In the present study, we show that ΔNp63α knockdown in SCC cells leads to elevated Rac1 activity and upregulation of the Rac-specific GEF PREX1. To our knowledge, this is the first study that implicates decreased activation of Rac1 in the anti-invasive role of ΔNp63α. This observation, together with the evidence that ΔNp63α inhibits cancer cell migration and invasion, provides critical mechanistic insight into the inhibitory role of p63 in SCC metastasis.

The small GTPase Rac1 dynamically regulates cytoskeletal organization, and therefore, acts as a major regulator of cell morphology, adhesion and migration. Rac1 hyperactivation is a hallmark of a variety of cancers, contributing to enhanced cancer cell migration, invasion, and metastasis [11, 12]. As such, Rac1 represents a potential therapeutic target for cancer metastasis. The mechanisms leading to elevated Rac1 activity in cancer are not fully understood, and to date, efforts to exploit Rac1as a therapeutic target in cancer have not been comprehensively examined. Our findings indicate that loss of ΔNp63α is permissive for Rac1-dependent invasion in SCC, and this fits with the loss of ΔNp63α in advanced metastatic disease [6, 9, 38].

Hyperactivation and/or overexpression of Rac-GEFs have been linked to aberrant activation of Rac1 in cancer [13]. However, due to the large complexity and context-specific Rac-GEF expression and regulation, there is a pressing need to dissect the upstream regulatory events as well as their expression control mechanisms in specific cancers. Our analysis identified PREX1 as a Rac1-GEF that is upregulated upon ΔNp63α knockdown in SCC cells. PREX1 is primarily expressed in hematopoietic cells, neurons, and endothelial cells, and its expression is low in most normal epithelial cells [17, 22, 39]. Previous studies revealed that PREX1 is prominently upregulated in multiple human cancers. Moreover, PREX1 has been causally linked to increased tumorigenesis and metastasis [17]. PREX1 is highly expressed in oral SCC (OSCC), and is associated with metastatic disease and poor prognosis. Amplification or epigenetic dysregulation of PREX1 may contribute to its overexpression in some human cancers [22, 40, 41]. We found that ΔNp63α suppresses the expression of PREX1 in cutaneous SCC cells (A431) as well as in JHU-006, JHU-029, and FaDu HNSCC cells, an effect that was observed both at mRNA and protein levels. The mechanism underlying ΔNp63α-mediated suppression of PREX1 was investigated using a PREX1 promoter-driven luciferase reporter, whose activity inversely correlated with ΔNp63α abundance but showed that PREX1 is not a direct target of ΔNp63α.

Endogenous PREX1 knockdown reduces Rac1 activity and cancer cell invasion in SCC cells. In mouse models, deletion of the *PREX1* gene leads to reduced Rac1 activity and metastasis in melanoma [19]. It has been well-established that PREX1-mediated activation of Rac1 promotes membrane ruffling and lamellipodia formation, which contributes to cell migration [21]. We showed that ΔNp63α inhibits PREX1-mediated activation of Rac1 by reducing the expression of PREX1, and that PREX1 knockdown abrogates the elevated SCC cell invasion observed as a consequence of ΔNp63α knockdown. Thus, loss of PREX1 represents a critical event for mediating the effect of ΔNp63α on Rac1 activation. We postulate that ΔNp63α is a promising therapeutic target for reducing the Rac-GEF/Rac1 metastatic signaling in cancer.

In summary, our study identified a novel mechanism by which ΔNp63α inhibits Rac1 activity and cancer cell invasion. ΔNp63α indirectly controls PREX1 promoter transcriptional activity and downregulates its expression, which in turn inhibits activation of Rac1 and reduces cancer cell invasion. These results may facilitate the development of novel, effective therapeutic approaches for metastatic cancer.

## Materials and methods

### Cell culture and reagents

A431 SCC, HaCaT non-tumorigenic immortalized human keratinocyte, FaDu HNSCC, and H1299 human non-small cell lung carcinoma cell lines were purchased from American Type Culture Collection (Manassas, Virginia, USA) and grown in Dulbecco’s modified Eagle medium (DMEM) supplemented with 8% fetal bovine serum (FBS), 250 U penicillin, and 250 μg streptomycin. The HNSCC cell lines JHU-006 and JHU-029 were a generous gift of Dr. James W. Rocco (Ohio State University, Columbus, Ohio, USA). JHU-006 and JHU-029 cells were grown in Roswell Park Memorial Institute (RPMI) Medium supplemented with 10% FBS, 250 U penicillin, and 250 μg streptomycin. NSC23766 [(N6-[2-[[4- (diethylamino)-1-methylbutyl]amino]-6-methyl-4-pyrimidinyl]-2-methyl-4,6-quinolinediamine trihydrochloride] was purchased from Sigma (St. Louis, MO).

### siRNA and DNA transfection

AllStars negative control, non-targeting control (NTC), and sip63 siRNAs used in this study were purchased from Qiagen (Valencia, CA, USA). PREX1 siRNA was purchased from Dharmacon (Lafayette, CO, USA). Cells were transfected using Lipofectamine RNAi-Max (Life Technologies, Carlsbad, CA, USA) or Lipofectamine 2000 (Invitrogen, Carlsbad, CA, USA) according to the manufacturer’s instructions, as reported previously [25]. Cells were harvested 24 or 48 hours after transfection. Resuspended cell pellets were used for immunoblotting, Rac1 pull-down assays, and luciferase assays.

### *PREX1* promoter cloning and luciferase reporter assay

The full-length 2024 bp *PREX1* promoter [22] was amplified by PCR using the forward primer 5′- CGGACTCGAGGCTCTCACAAAGACTCCCCTTTT-3′ and the reverse primer 5′-CCCAAGCTTGCTCCTTCCGTCGCGCCGAG-3′ and the PCR product was inserted into the *XhoI* and *HindIII* cloning sites in the pGL3-basic vector, which carries the luciferase reporter gene (pGL3-basic-Luc, Promega, Madison, WI, USA). A 229 bp fragment of the *PREX1* promoter containing a putative p63 binding site (chr20:48828385-48828404) [30, 31] was PCR-amplified using forward primer 5′-CGGACTCGAGCTCCGCAGCGAGCTTTCCCAGCCC-3′ and the reverse primer 5′-CCCAAGCTTCCGGAAGGGCCCCGCGGAGCC-3 and the PCR product was inserted into the *XhoI* and *Hin*d*III* cloning sites in pGL3-basic-Luc to generate ΔPREX1-Luc. The wild-type p63 binding site in ΔPREX1-Luc (CTGCAAGCAGGAGCCTGCGC) was mutated to CTGAAAACAAGAAACTACGC in the mutant binding site plasmid (mut-BS) (GenScript, Piscataway, NJ, USA).

For overexpression studies, H1299 cells were co-transfected with empty vector (EV) or expression plasmid encoding ΔNp63α and *PREX1* or Δ*PREX1* luciferase reporter plasmids. For knockdown studies, JHU-006 cells were transfected with non-targeting control (NTC) and sip63 siRNAs. After 24 hours, cells were transfected with a plasmid encoding either *PREX1* or Δ*PREX1* luciferase reporter. Cells were transfected with a *Renilla* luciferase expression plasmid to estimate transfection efficiency and normalize experimental data. After transfection, cells were harvested in passive lysis buffer, and cell lysates were used for dual luciferase assay, according to the manufacturer’s instructions (Promega, Madison, WI). Relative luciferase unit (RLU) values were calculated from the ratio of Firefly luciferase activity to *Renilla* luciferase activity and normalized to luciferase activity in cells co-transfected with empty pLG3 basic vector plasmid.

### Western blot

For immunoblotting, cells were lysed in buffer containing 50 mM Tris-HCl pH 8, 120 mM NaCl, 5 mM sodium pyrophosphate phosphatase inhibitor, 10 mM NaF, 30 mM paranitrophenylphosphate, 1 mM benzamidine, 0.1% NP-40, 1% Triton X-100, 0.2 mM PMSF, 100 nM sodium orthovanadate, and 10% protease inhibitor cocktail (Sigma, St. Louis, MO). Immunoblotting was carried out as previously described [25]. Proteins were detected using the following antibodies: rabbit polyclonal anti-p63 [N2C1] (Gene Tex, Irvine, CA, USA), mouse monoclonal anti-Rac1 [23A8] (Abcam, Cambridge, MA, USA), mouse monoclonal anti-β-actin antibody from Santa Cruz Biotechnology (Santa Cruz, CA, USA), and rabbit polyclonal anti- PREX1 (Sigma, St. Louis, MO). Horseradish peroxidase-conjugated secondary antibody (Promega, Madison, WI, USA) was used for chemiluminescence detection with the Western Lightning Plus kit (Perkin Elmer, Waltham, MA, USA).

### Cell invasion assay

Cell invasion was assessed using a two-chamber transwell system. A total of 5.0 × 10^4^ transiently transfected JHU-006 cells were suspended in serum-free medium, seeded into 8 μm pore size inserts (BD Biosciences) coated with 1 mg/mL Matrigel (BD Biosciences), and placed into the well of a 24-well plate. Then, RPMI containing 10% FBS was added to the bottom of each insert and incubated for 21 h. Cells that did not invade were removed using a cotton swab. Invading cells attached to the bottom of the transwell were fixed with 4% of paraformaldehyde and washed once with Dulbeco’s PBS. Cells were stained with 0.1% crystal violet and imaged in 4-6 random fields at 100× magnification using a Leica CTR 6000 Microscope (Leica Microsystems, Wetzlar, Germany) and ImagePro 6.2 software (Media Cybernetics, Bethesda, MD). Cells were counted manually and the average number of cells per field was calculated.

### RNA isolation and TaqMan qRT-PCR

Total RNA was extracted using the EZNA RNA isolation kit according to the manufacturer’s instructions (Omega Bio-Tek, Norcross, GA, USA). Quantitative RT-PCR was carried out as previously described using the Applied Biosystem 7900HT or QuantStudio 7 Flex Real-Time PCR Systems and Assays on Demand^TM^ (AOD) for GAPDH (4325792) and *PREX1* (Hs01031507_m1). Data were normalized to endogenous GAPDH (Life Technologies, Carlsbad City, CA, USA) [42, 43]. qRT-PCR reactions were run in triplicate. Data were analyzed using the 2^−ΔΔCT^ method [44] and statistical significance was analyzed using two-tailed Student’s unpaired *t* test.

### Rac1-GTP pull-down assay

Rac1-GTP was quantified using a Rac1 pull-down activation assay kit (Cytoskeleton, BK035, Denver, CO), in which PAK-PBD is fused to GST and glutathione affinity beads are used to isolate and quantify PAK-PBD-bound proteins. Assays were performed 48 h after transfection with siRNA or 24 h after transfection with overexpression plasmid DNA. Briefly, cells were lysed in ice-cold lysis buffer (50 mM Tris pH 7.5, 10 mM MgCL2, 0.5 M NaCl, and 2% Igepal), containing 1× protease inhibitor cocktail. Cell lysates were immediately clarified by centrifugation at 10,000 × *g* for 1 min at 4°C. Protein concentrations were determined by BCA assay (Thermo Fisher Scientific Inc., Fremont, CA, USA). Equivalent concentrations of protein (300–500 μg) were added to 10 μL PAK-PBD beads and rotated at 4 °C on a tube rotator for 1 h. The PAK-PBD beads were pelleted by centrifugation at 5000 × *g* at 4 °C for 3 min, washed twice with 500 μL wash buffer, and resuspended in 20 μL wash buffer. Rac1-GTP-bound PAK-PBD beads were loaded on a 10% SDS-PAGE gradient gel, followed by immunoblot analysis using mouse monoclonal anti-Rac1 (23A8) to detect total Rac1.

### GST-Rac1 fusion beads and immunoprecipitation assay

Plasmids expressing recombinant wild-type (WT) and G15A GST-Rac1 were obtained from Dr. Garcia-Mata (University of Toledo). Recombinant proteins were expressed and purified as previously described [32]. Recombinant proteins were incubated with 500 μL pre-equilibrated Glutathione-Sepharose 4B beads (Sigma-Aldrich) at 4 °C for 1 h. After incubation, the samples were centrifuged, and pelleted beads were washed once with lysis buffer and twice with 20 mM HEPES pH 7.5, 150 mM NaCl (HBS) containing 5 mM MgCl_2,_ and 1 mM DTT. Washed beads were resuspended in an aliquot of the supernatant, 250 μL glycerol was added, and the resuspended beads were diluted in HBS containing 5 mM MgCl_2_, 1 mM DTT, and glycerol to a final total protein concentration of 1–3 mg/mL.

For immunoprecipitation, cells were lysed in NP-40 lysis buffer (50 mM Tris-HCl pH 8, 150 mM NaCl, 5 mM EDTA pH 8, 1% NP-40, 2 mM DTT, and 2 mM PMSF) containing protease inhibitor cocktail (Sigma-Aldrich). Whole-cell extracts (1 mg) were added to 20 μg WT or G15A GST-Rac1 beads and rotated at 4°C for 1 h. After centrifugation, pelleted beads were washed three times with wash buffer (0.05% Tween-20 in 1× PBS) and resuspended in 20 μL wash buffer. Immunoprecipitated protein complexes were resolved on a 7.5% SDS-PAGE gradient gel followed by immunoblot analysis.

### Statistical analysis

Data are presented as mean ±1 standard deviation (SD) or Standard Error of the Mean (SEM). Statistical significance between groups was determined using Student’s unpaired *t* test or mixed effects ANOVA (where indicated). Statistically significant values (*P* ≤ 0.05) are indicated with an asterisk or the # symbol.

### Supplementary information

Supplemental Figures

Supplementary western blot images

## Data Availability

The data used to support the findings of this study are available from the corresponding author upon request.
