# Supplementary material for: ∆Np63α inhibits Rac1 activation and cancer cell invasion through suppression of PREX1
Source: Cell Death Discov. 2024 Jan 8;10:13. doi: 10.1038/s41420-023-01789-0 (PMC10774331; doi:10.1038/s41420-023-01789-0)
Supplement: Supplementary file 1 — Supplemental Figures [file 41420_2023_1789_MOESM1_ESM.pdf]

Supplemental Figures

ΔNp63a inhibits Rac1 activation through suppression of *PREX1* leading to inhibition in cancer cell invasion

Amjad A. Aljagthmi[1], Akshay Hira[1], Jin Zhang[1], Mariana Cooke[2], Marcelo G. Kazanietz[2], Madhavi P. Kadakia[1]

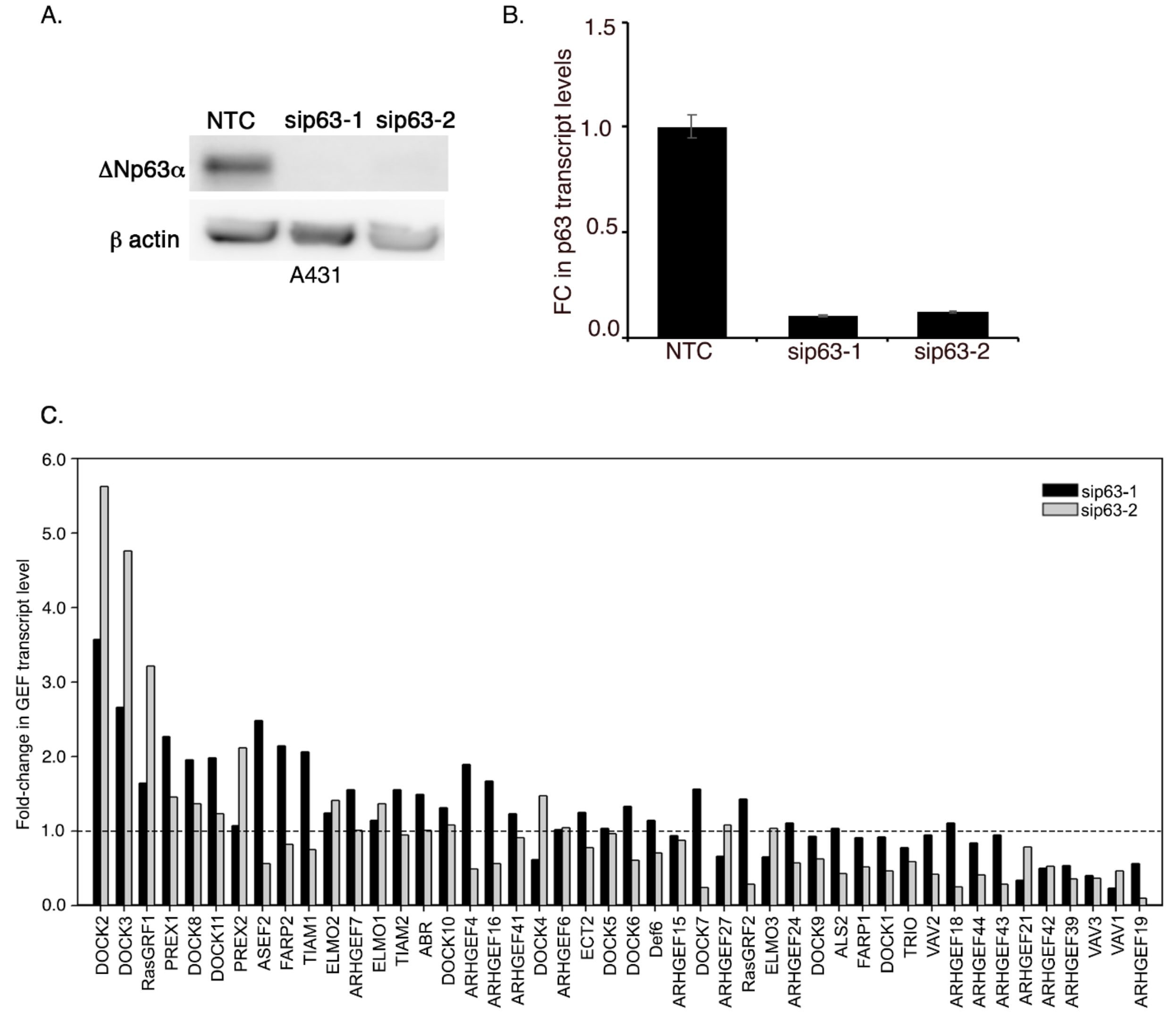

Supplemental Figure 1. Effect of ΔNp63α knockdown on expression of 43 Rac-GEFs. A431 cells were transfected with non-targeting control (NTC) or two different p63-targeted siRNAs, sip63-1 or sip63-2. **(A)** Immunoblots of ΔNp63α in A431 samples used in the Rac-GEF array. **(B)** Total RNA was extracted and ΔNp63α transcript levels were measured by TaqMan based qRT-PCR. Data are presented as mean ±1 SD. **(C)** Transcription of Rac-GEFs in A431 cells transfected with NTC, sip63-1 or sip63-2 as determined by GEF Array. Transcripts for 43 Rac-GEFs were quantified by qRT-PCR in 96-well plates and normalized to beta-2-microglobulin and ubiquitin C (UBC). The fold-change in each transcript relative to NTC-transfected cells was calculated for sip63-1 (black bars) or sip63-2 (gray bars).

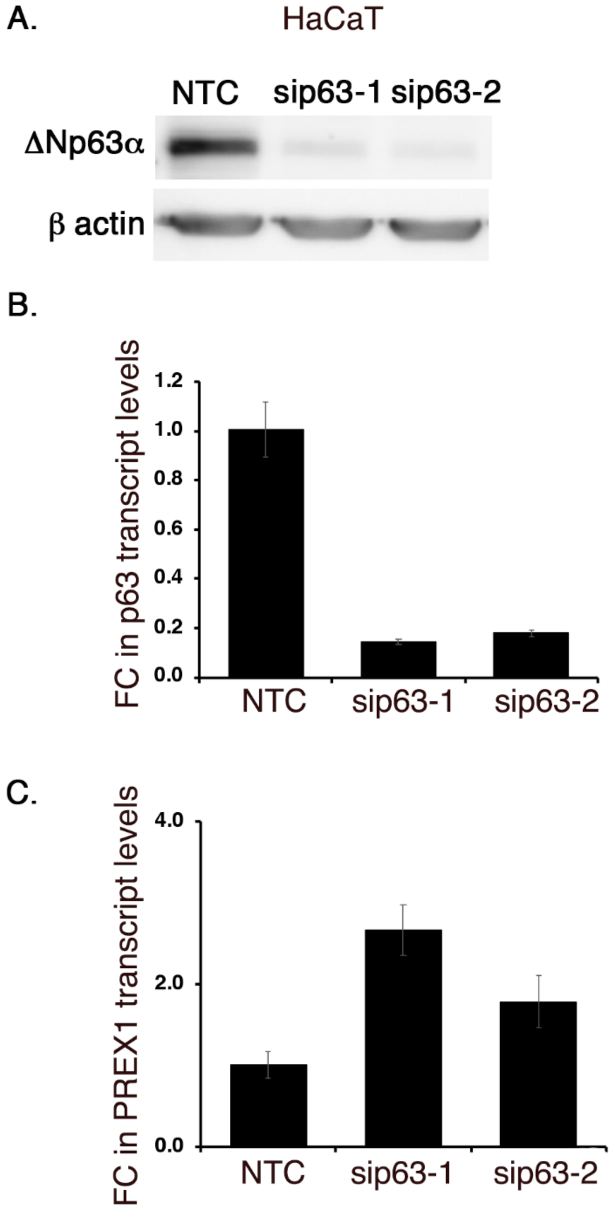

Supplemental Figure 2. ΔNp63α knockdown upregulates *PREX1* transcript in HaCaT cells. HaCaT cells were transfected with non-targeting control (NTC) or two different p63-targeted siRNAs, sip63-1 or sip63-2. **(A)** Representative immunoblot of p63 in HaCaT cells transfected. Total RNA was extracted and **(B)** ΔNp63α or **(C)** *PREX1* mRNA was quantified using Taqman qRT-PCR. The fold-change (FC) in ΔNp63α and *PREX1* mRNA was calculated in sip63-1 and sip63-2 transfected cells relative to NTC-transfected cells. Error bars indicate +1 SD from 3 independent experiments.

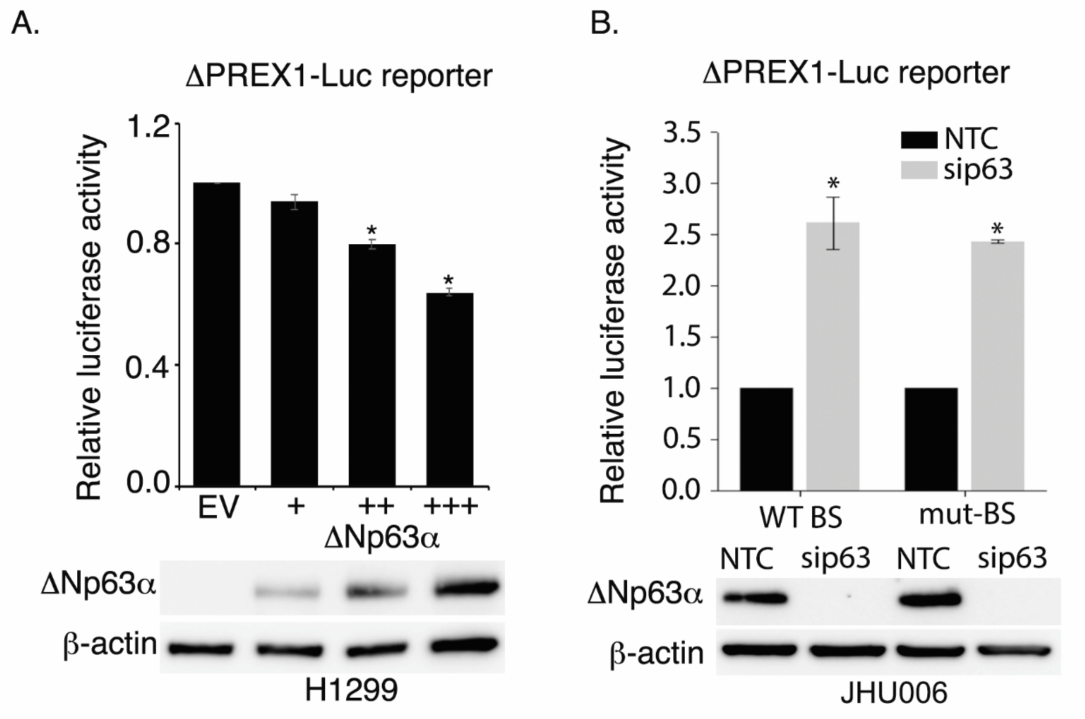

Supplemental Figure 3. ΔNp63α indirectly represses the transcription activity of *PREX1* promoter. (A) H1299 cells were co-transfected with Δ*PREX1*-Luc reporter plasmid and either empty vector (EV) control or increasing concentrations of ΔNp63α expression plasmid. At 24 h after transfection, a dual luciferase assay was performed in triplicate. Relative luciferase units (RLU) were calculated as the ratio of Firefly luciferase activity to *Renilla* luciferase activity and normalized to EV control. Values are shown as mean ± S.E.M from 3 independent experiments. Statistically significant values ($P \leq 0.05$) relative to EV controls are indicated with an asterisk. **(B)** JHU006 cells were co-transfected with nontargeting control (NTC) siRNA or p63-targeted siRNA (sip63). After 24 h, cells were transfected with Δ*PREX1*-Luc with the wild-type p63 binding site (WT BS) or mutant Δ*PREX1*-Luc reporter plasmid (mut-BS). Following another 24h, a dual luciferase assay was performed. RLU were calculated as the ratio of Firefly luciferase activity to *Renilla* luciferase activity and normalized to NTC control. Values are shown as mean ±1 S.E.M from 2 independent experiments. Statistically significant values ($P \leq 0.05$) relative to NTC control are indicated with an asterisk. ΔNp63α protein and the β-actin loading control were analyzed by immunoblot (bottom).
