# Supplementary figures and images for: ∆Np63α inhibits Rac1 activation and cancer cell invasion through suppression of PREX1
Source: Cell Death Discov. 2024 Jan 8;10:13. doi: 10.1038/s41420-023-01789-0 (PMC10774331; doi:10.1038/s41420-023-01789-0)

Figure 1A

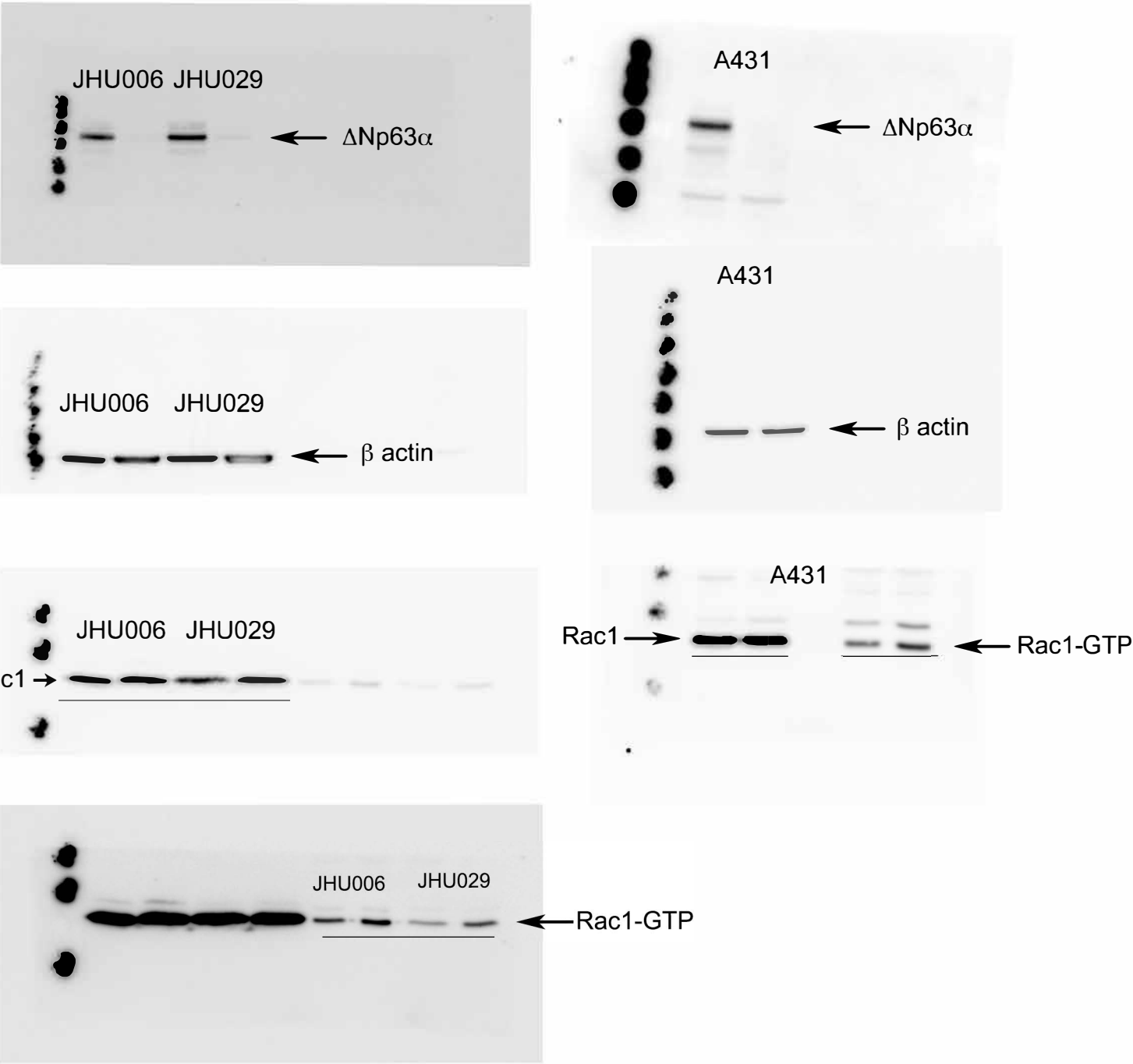

Figure 1B

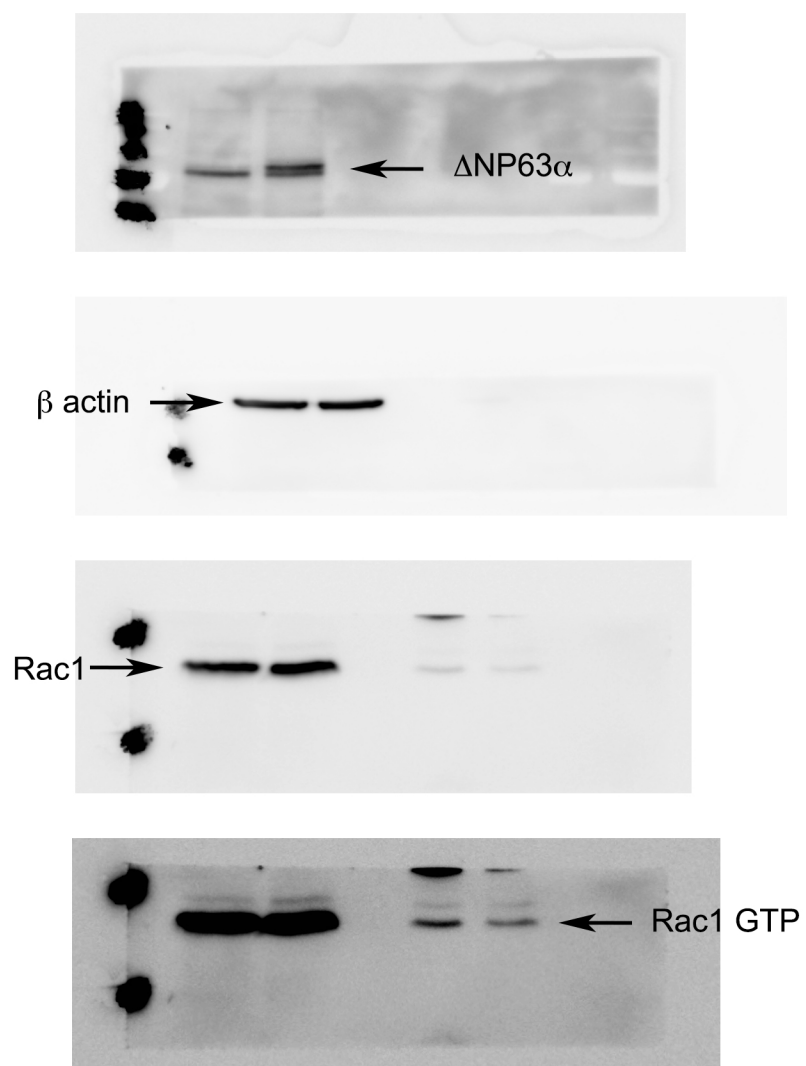

Figure 2A

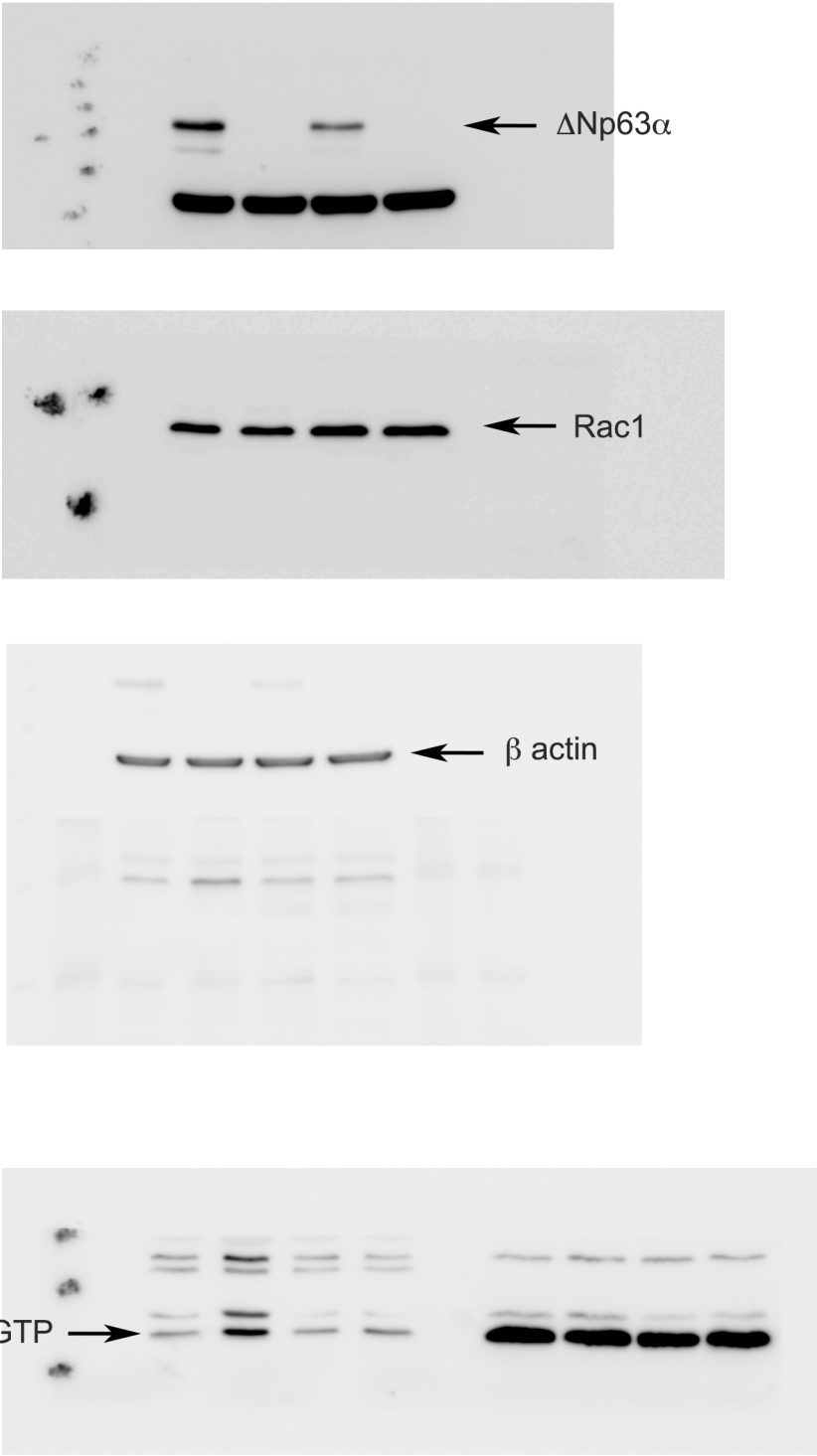

Figure 3A

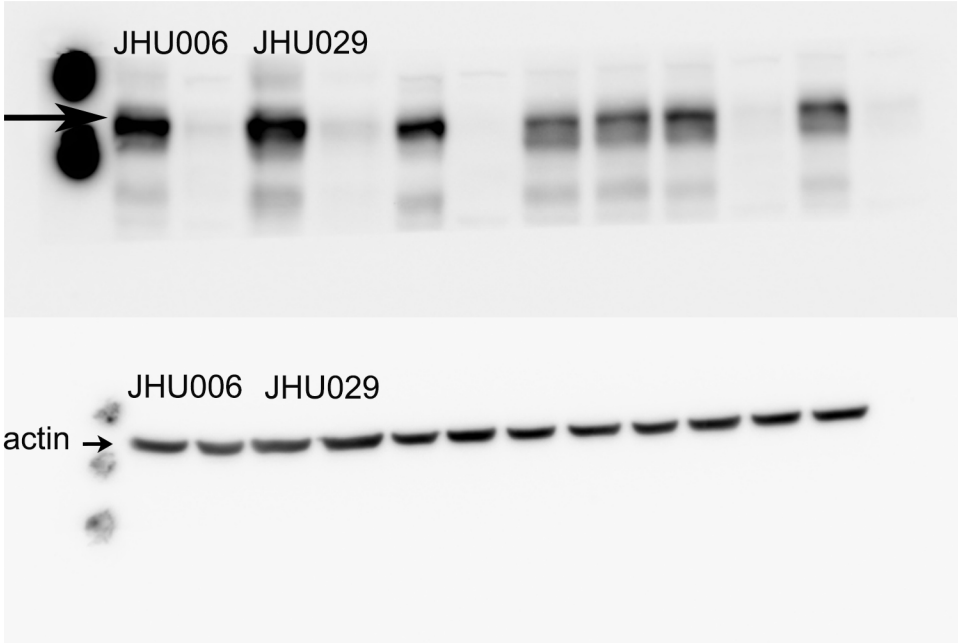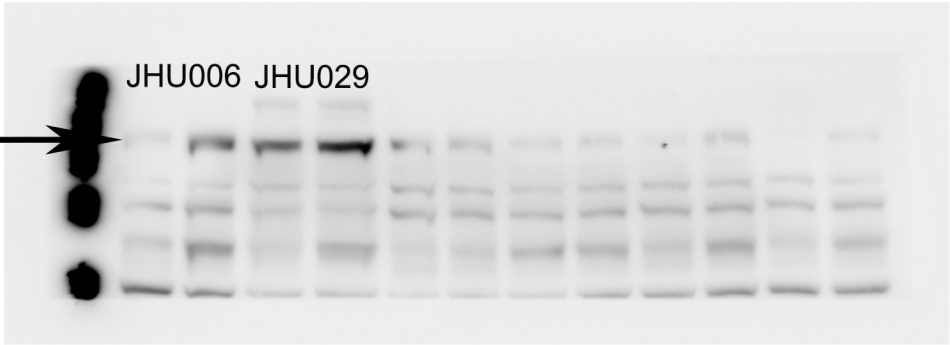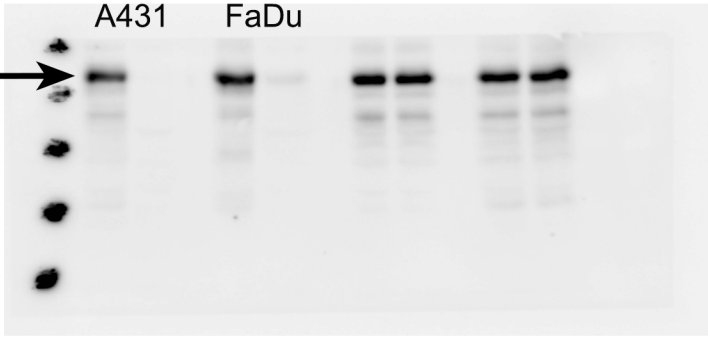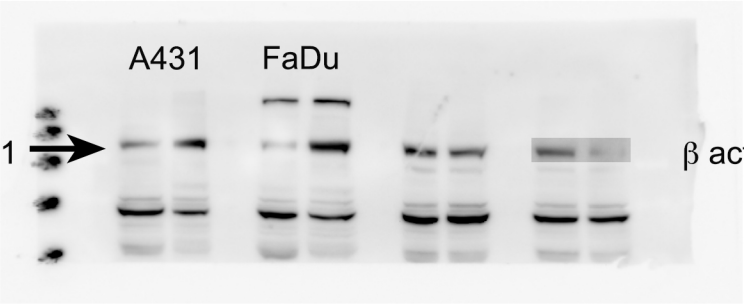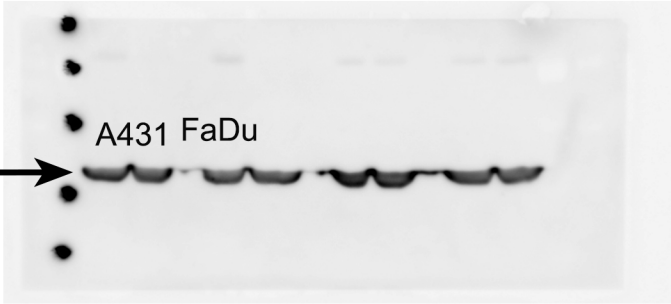

Figure 3B

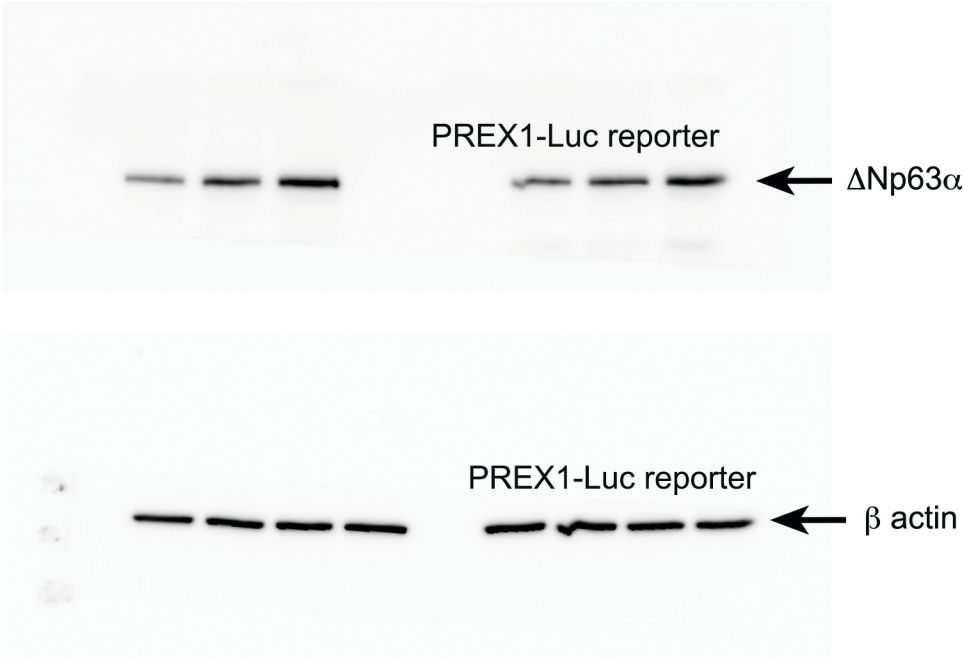

Figure 3C

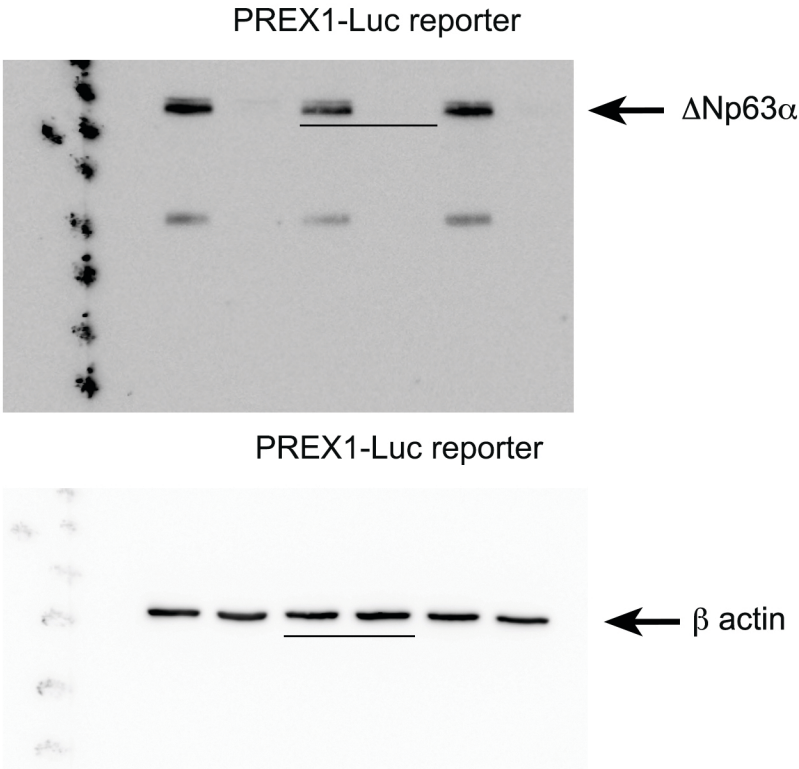

Figure 4A

For the IP:

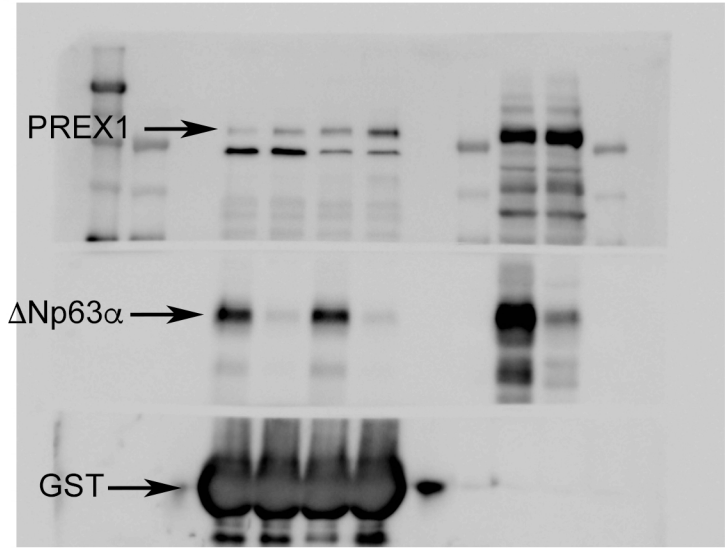

For the input:

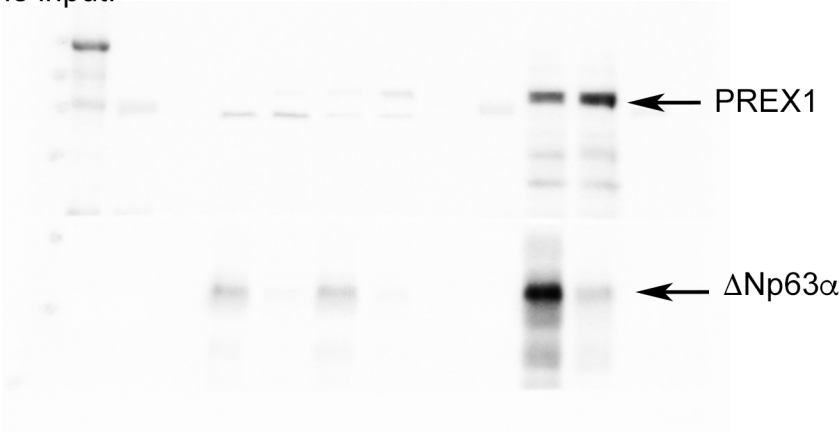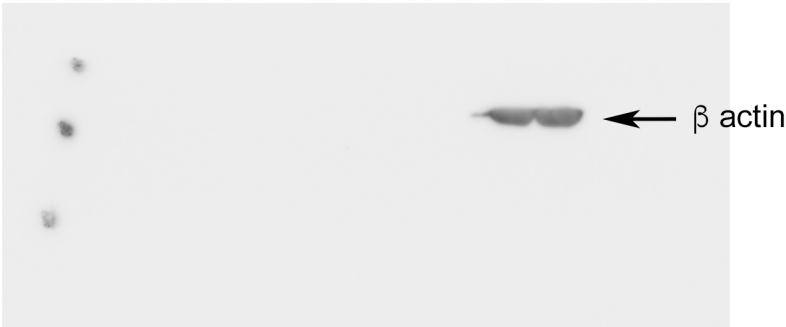

Figure 5A

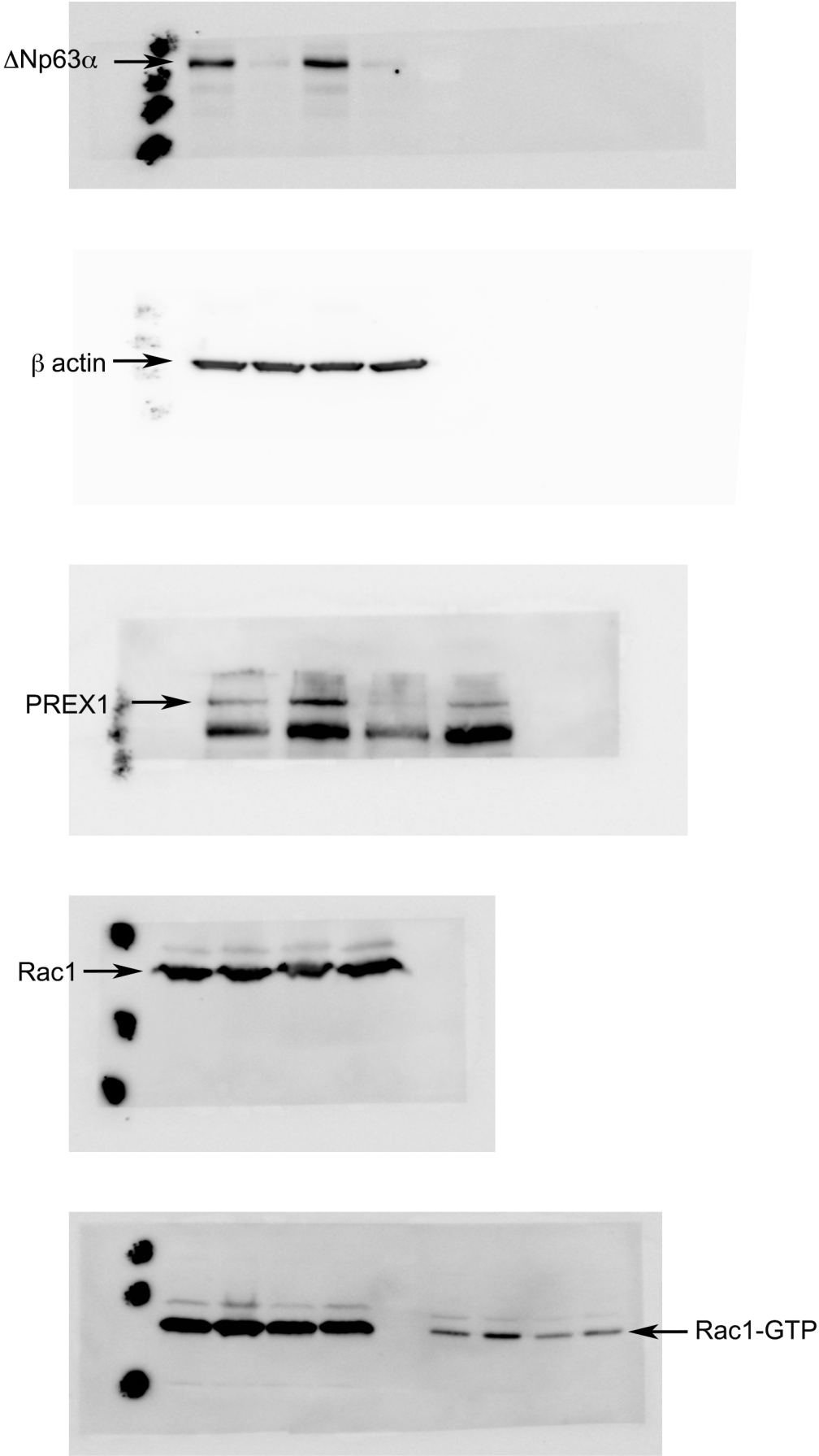

Supplement: Supplementary file 2 — Supplementary western blot images [file 41420_2023_1789_MOESM2_ESM.pdf]
